# OR11H1 Missense Variant Confers the Susceptibility to Vogt‒Koyanagi‒Harada Disease by Mediating Gadd45g Expression

**DOI:** 10.1002/advs.202306563

**Published:** 2024-01-02

**Authors:** Xingran Li, Guoqing Wang, Xiaotang Wang, Wanqian Li, Na Li, Xianyang Liu, Wei Fan, Siyuan He, Yue Han, Guannan Su, Qingfeng Cao, Peizeng Yang, Shengping Hou

**Affiliations:** ^1^ Chongqing Branch of National Clinical Research Center for Ocular Diseases; Chongqing Key Laboratory of Ophthalmology; Chongqing Eye Institute The First Affiliated Hospital of Chongqing Medical University Chongqing 400042 China; ^2^ Department of Laboratory Medicine Beijing Tongren Hospital, Capital Medical University Beijing 100005 China; ^3^ Beijing Novogene Bioinformatics Technology Co.,Ltd Beijing 100600 China; ^4^ Beijing Institute of Ophthalmology Beijing Tongren Eye Center Beijing Ophthalmology & Visual Sciences Key Laboratory Beijing Tongren Hospital Capital Medical University Beijing 100730 China

**Keywords:** GADD45G, OR11H1, RPE, uveitis, Vogt‒Koyanagi‒Harada disease, whole‐exome sequencing

## Abstract

Vogt‒Koyanagi‒Harada (VKH) disease is a severe autoimmune disease. Herein, whole‐exome sequencing (WES) study are performed on 2,573 controls and 229 VKH patients with follow‐up next‐generation sequencing (NGS) in a collection of 2,380 controls and 2,278 VKH patients. A rare c.188T>C (p Val63Ala) variant in the olfactory receptor 11H1 (OR11H1) gene is found to be significantly associated with VKH disease (rs71235604, P_combined_ = 7.83 × 10^−30^, odds ratio = 3.12). Functional study showes that OR11H1‐A63 significantly increased inflammatory factors production and exacerbated barrier function damage. Further studies using RNA‐sequencing find that OR11H1‐A63 markedly increased growth arrest and DNA‐damage‐inducible gamma (GADD45G) expression. Moreover, OR11H1‐A63 activates the MAPK and NF‐κB pathways, and accelerates inflammatory cascades. In addition, inhibiting GADD45G alleviates inflammatory factor secretion, likely due to the regulatory effect of GADD45G on the MAPK and NF‐κB pathways. Collectively, this study suggests that the OR11H1‐A63 missense mutation may increase susceptibility to VKH disease in a GADD45G‐dependent manner.

## Graphical abstract

A whole‐exome sequencing study with follow‐up next‐generation sequencing in controls and VKH patients is conducted. A rare c.188T>C (p.Val63Ala) variant in the olfactory receptor 11H1 (OR11H1) gene is found to be significantly associated with VKH disease in Chinese Han population. Functional studies show that the OR11H1‐A63 missense mutation may increase susceptibility to VKH disease in a GADD45G‐dependent manner.

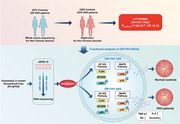

## 1 Introduction

Vogt‒Koyanagi‒Harada (VKH) disease is an autoimmune disease that threatens vision. It is common in China and Japan, and is rarely found in the United States of America and the United Kingdom.^[^ ^1^, ^2^, ^3^ ^]^ VKH disease selectively involves the uvea, central nervous system, inner ear, skin, and other tissues containing melanocytes, resulting in sudden uveitis, white eyebrows, white hair, and leukoderma.^[^ ^4^, ^5^ ^]^ The retinal pigment epithelium (RPE) is a monolayer of pigment cells that is involved in the pathological process of VKH disease.^[^ ^6^, ^7^, ^8^, ^9^ ^]^ Long‐held theories suggest that the symptoms of VKH disease may be caused by an autoimmune response to melanin.^[^ ^4^, ^7^ ^]^ In the early stage of VKH disease, human melanoma cells are severely affected by T cells, presenting as folds of RPE.^[^ ^10^ ^]^ In the chronic phase of VKH disease, proliferated RPE cells constitute the Dalen–Fuchs nodule, a typical histologic feature in this disease.^[^ ^11^ ^]^ Moreover, RPE cells attract inflammatory cells to the retina by secreting IL‐6, iNOS, TNF‐α, and other inflammatory cytokines.^[^ ^12^, ^13^, ^14^ ^]^ Many studies have used LPS‐stimulated RPE cells to study the function of RPE in uveitis.^[^ ^15^, ^16^ ^]^ Furthermore, the RPE is responsible for the constitutive phagocytosis of shedding outer segments, the provision of nutrients to the retina, and the transportation of waste products between the retinal and choroidal blood flow.^[^ ^17^ ^]^ Therefore, assessing changes in the RPE is essential to further elucidate the pathogenesis and facilitate the treatment of VKH disease.

Although the etiology and pathogenesis of VKH disease remain unclear, it has been reported to be related to genetic and immunological factors.^[^ ^18^ ^]^ Previous genetic studies have found several genes associated with VKH disease, including IL23R‐C1orf141, ADO‐ZNF365‐EGR2, IL17F, CTLA4, and MIF.^[^ ^19^, ^20^, ^21^, ^22^ ^]^ Human leukocyte antigens (HLAs), such as HLA‐DRB1 ^*^ 0405, DRB1 ^*^ 0401, and HLA‐B ^*^ 51, are strongly associated with VKH disease or Behcet's disease in various ethnic populations.^[^ ^4^, ^23^, ^24^, ^25^, ^26^, ^27^, ^28^ ^]^ However, HLA genes are only estimated to account for ≈20% of the overall genetic susceptibility to VKH disease.^[^ ^19^ ^]^ The identified HLA variants are mainly noncoding variants or nonfunctional variants, and it is very intricate to study the specific functional roles of HLA genes. Thus, we focused on the association of non‐HLA genes with VKH disease in this study. Although exons (protein‐coding regions) account for only 1% of the human genome, 85% of disease‐causing mutations in the human genome are in exonic regions.^[^ ^19^, ^29^ ^]^ Therefore, we performed a whole‐exome sequencing (WES) study to complement the genome‐wide association study (GWAS) by examining the direct relationship between 2573 controls and 229 VKH patients of Han Chinese descent. We identified that a variant in the olfactory receptor 11H1 (OR11H1) gene was significantly associated with VKH disease. ORs (olfactory receptors) are members of the G protein‐coupled receptor (GPCR) family, which is one of the largest gene families in humans.^[^ ^30^, ^31^ ^]^ It has been shown that more than 580 ORs are expressed in macrophages.^[^ ^32^ ^]^ In addition, the expression of ORs such as ORL622, Olfr2, and OR6A2 was increased after LPS stimulation in macrophages, which resulted in the transcription of inflammasome components.^[^ ^33^ ^]^ There is no study on the expression of OR11H1 and its function following LPS stimulation. Moreover, it is not known which subtypes of ORs promote or modulate inflammation. Whether ORs can improve devastating ocular inflammation is still controversial.

In the following RNA sequencing, we identified that OR11H1‐A63 increased the gene expression of growth arrest and DNA‐damage‐inducible, gamma (GADD45G) in ARPE‐19 cells. GADD45 family members are known as stress sensors, and they can be rapidly induced by environmental toxins and inflammatory cytokines.^[^ ^34^, ^35^ ^]^ GADD45s include GADD45A, GADD45B, and GADD45G, which are associated with the regulation of cell survival, apoptosis, inflammation, and cell cycle arrest.^[^ ^36^ ^]^ Among these, GADD45G, the tumor suppressor gene, was found to have an effect on the inflammatory response. GADD45G can regulate the JAK‐STAT3 pathway to inhibit hepatocellular carcinoma.^[^ ^37^ ^]^ GADD45G is reported to promote cardiomyocyte apoptosis in a p38 MAPK‐dependent manner.^[^ ^38^ ^]^ However, whether GADD45G plays an important regulatory role in VKH disease remains completely unknown.

In this study, WES analysis showed that the OR11H1 (p.Val63Ala) missense variant conferred susceptibility to VKH disease in the Han Chinese population. Further functional studies showed that OR11H1‐A63 increased inflammatory cascades and activated the MAPK and NF‐κB pathways in ARPE‐19 cells in a GADD45G‐dependent manner. Our results suggested that OR11H1 might be a disease‐causing gene for VKH disease, providing a novel target and new strategy for the treatment of uveitis in the future.

## 2 Results

### 2.1 Recruitment of VKH Patients and Normal Controls

WES was used to sequence the exomes, which after quality control captured sequence data for 211419 exomes from 19111 genes in both 229 cases and 2573 controls. To obtain mutations that influence gene expression products (functional), the mutation sites of well‐known datasets (dbSNP, 1000 Genome, and HapMap) were screened, and synonymous mutations were excluded. A total of 51 independently associated SNPs were selected from the WES stage, and the associations of each SNP were then screened and typed by next‐generation sequencing (NGS) from 2278 VKH patients and 2380 controls. The variants in the WES and NGS controls match those in previous population studies. After filtering out the SNPs that did not meet the conditions, 39 SNPs were obtained. SNPs with HWE less than 0.05 and P value more than 0.05 were screened out after combining the results of two sequencing, and 8 SNPs strongly associated with VKH disease were obtained (**Table** **1**, Table S1, Supporting Information). All VKH patients and controls enrolled in this study were from a Han Chinese population.

**Table 1:** Summary of SNPs significantly associated with VKH disease in WES and replication studies.

Genes	SNPs	Chr	MA	Stage	MAF	HWE	*P* value	OR	95%CI
					(case/control)				
HLA‐DRA	rs7192	6	T	WES	0.096/0.262	0.11	3.89E‐15	0.30	0.22‐0.41
				Replication	0.115/0.252	0.40	1.88E‐64	0.39	0.35‐0.43
				Combined	0.113/0.257		3.05E‐92	0.37	0.34‐0.41
HLA‐DQA2	rs2071800	6	A	WES	0.100/0.039	0.43	1.07E‐09	2.73	1.95‐3.82
				Replication	0.094/0.048	0.01	5.06E‐18	2.05	1.74‐2.42
				Combined	0.095/0.044		3.32E‐35	2.30	2.01‐2.63
NOTCH4	rs2071282	6	A	WES	0.227/0.039	0.80	8.70E‐65	7.23	5.57‐9.38
				Replication	0.224/0.041	0.99	1.98E‐151	6.73	5.74‐7.89
				Combined	0.225/0.040		8.03E‐271	6.94	6.15‐7.82
TNXB	rs2269428	6	T	WES	0.146/0.032	1.00	3.92E‐32	5.20	3.85‐7.04
				Replication	0.158/0.037	0.29	2.37E‐86	4.90	4.13‐5.82
				Combined	0.157/0.034		1.21E‐156	5.24	4.59‐5.98
TNXB	rs61744970	6	C	WES	0.146/0.032	1.00	6.36E‐32	5.17	3.82‐7.00
				Replication	0.157/0.036	0.21	1.41E‐83	4.95	4.15‐5.91
				Combined	0.156/0.034		1.48E‐154	5.26	4.60‐6.01
TNXB	rs78493656	6	T	WES	0.146/0.032	1.00	6.36E‐32	5.17	3.82‐7.00
				Replication	0.158/0.037	0.28	8.13E‐87	4.93	4.15‐5.85
				Combined	0.157/0.034		4.56E‐157	5.24	4.59‐5.99
PRRC2A	rs75044749	6	G	WES	0.094/0.018	0.59	1.85E‐23	5.51	3.79‐8.01
				Replication	0.099/0.021	0.30	1.67E‐57	5.19	4.16‐6.48
				Combined	0.099/0.020		1.02E‐104	5.49	4.63‐6.51
**OR11H1**	**rs71235604**	**22**	**G**	**WES**	**0.050/0.005**	**1.00**	**3.21E‐24**	**11.53**	**6.37‐20.85**
				**Replication**	**0.047/0.028**	**0.17**	**9.29E‐07**	**1.73**	**1.39‐2.16**
				**Combined**	**0.047/0.016**		**7.83E‐30**	**3.12**	**2.54‐3.83**

Chr., chromosome; MA, minor allele; MAF, minor allele frequency; HWE, Hardy‐Weinberg Equilibrium; OR: odds ratio for the minor allele; 95% CI, 95% confidence intervals.

### 2.2 rs71235604 in the OR11H1 Gene was Associated with VKH Disease

**Figure 1:**
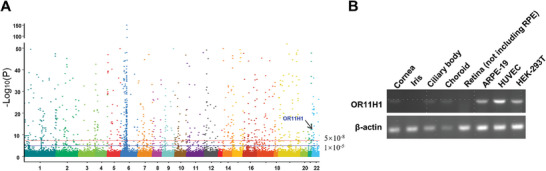
rs71235604 in the OR11H1 gene was associated with VKH disease. A) Manhattan plot of P values on the ‐log_10_ scale for all SNPs in the whole‐exome sequencing (WES) stage. The red line represents P = 5 × 10^−8^, and the blue dashed line represents P = 1 × 10^−5^. B) OR11H1 mRNA expression in different human tissues and cell lines by semiquantitative PCR.

PCA and Q‒Q plots indicated that the VKH patients and controls enrolled in this study were of Han Chinese descent and well matched, suggesting that the impact of population stratification on the genetic association in the enrolled samples is negligible (Figure S1, Supporting Information).^[^ ^39^ ^]^ We identified 3011 SNPs exceeding a genome‐wide significance level (P < 5 × 10^−8^, **Figure** **1**A). Eight SNPs were found to be greatly associated with VKH disease after NGS. Notably, only one of the eight SNPs was found on Chr 22, the others were all found on Chr 6. As Chr 6 is known to contain human leucocyte antigen (HLA) genes, our results identified several HLA susceptibility loci for this disease, including HLA‐DRA, HLA‐DQA2, NOTCH4, TNXB, and PRRC2A. Numerous studies have examined the association between HLA and VKH disease, therefore, we focused on the non‐HLA gene OR11H1 in the current study.^[^ ^4^, ^23^, ^24^, ^25^ ^]^ The OR11H1 rare variant (a frequency less than 0.05) in Chr 22 rs71235604 (c.188T>C) was significantly associated with VKH disease (WES: P = 3.21 × 10^−24^, OR = 11.53; NGS: P = 9.29 × 10^−7^, OR = 1.73; Combined: P = 7.83 × 10^−30^, OR = 3.12) (Table 1). The Human Protein Atlas website was used to assess the expression of OR11H1, and the results showed that there was no detected expression in immune cells (Figure S2, Supporting Information). Next, we investigated the expression of OR11H1 mRNA in intraocular tissues and cells that are involved in the uveitis pathogenesis. The results showed that OR11H1 was expressed in ARPE‐19, HUVEC, 293T cell, cornea, ciliary body, and choroid (**Figure** 1B). ARPE‐19 cells were chosen for follow‐up experiments since they are one of the key cells in the pathogenesis of VKH disease and pigment is thought to play an antigenic role in VKH development.^[^ ^8^, ^10^ ^]^ These results suggested that OR11H1 might play an important functional role in the development of uveitis.

### 2.3 OR11H1‐A63 Accelerated Inflammation and Barrier Breakdown in ARPE‐19 Cells

**Figure 2:**
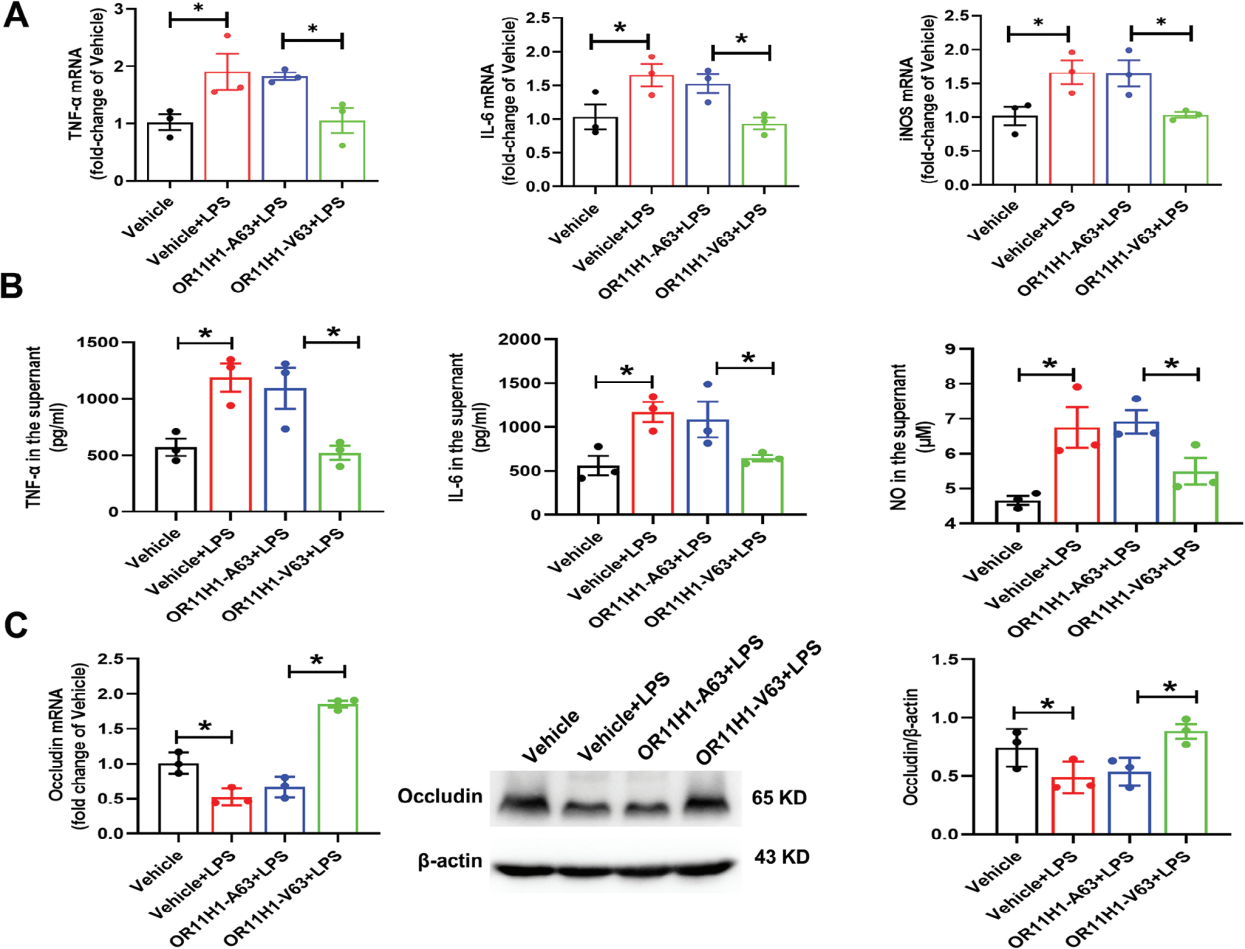
OR11H1‐A63 accelerated inflammation and barrier breakdown in ARPE‐19 cells. **A**RPE‐19 cells were transfected with OR11H1‐V63 and OR11H1‐A63 lentivirus, and the cells were randomly divided into the Vehicle, Vehicle + LPS, OR11H1‐A63 + LPS, and OR11H1‐V63 + LPS groups. After incubation with LPS (1 µg ml^−1^) for 24 h, the cells were collected. A,B) The levels of inflammatory factors, including TNF‐α, IL‐6, and iNOS/NO, were measured by RT‒qPCR, ELISA, and Griess reagent, respectively. C) The expression levels of Occludin (representative barrier marker) were measured by RT‒qPCR and Western blotting. ^*^ *P* < 0.05 by one‐way ANOVA (n = 3).

To elucidate whether the identified risk allele in OR11H1 contributed to the development of uveitis, we investigated the role of OR11H1‐A63 in ARPE‐19 cells. We transfected OR11H1‐V63 and OR11H1‐A63 lentivirus into ARPE‐19 cells. We first measured the overexpression efficiency by RT‒qPCR and Western Blotting. OR11H1‐V63/A63 extremely significantly overexpressed OR11H1 mRNA levels and OR11H1 protein levels (Figure S3, Supporting Information). As shown in **Figure** **2**A,B, OR11H1‐A63 significantly increased the expression of TNF‐α, IL‐6 and iNOS/NO compared with OR11H1‐V63 following LPS stimulation (Figure 2A,B). Furthermore, OR11H1‐A63 attenuated Occludin expression compared with OR11H1‐V63 following LPS stimulation (Figure 2C). The results showed that OR11H1‐V63 exerts protective effects on inflammatory response and barrier‐breaking processes, while OR11H1‐A63 loses this effect and leads to the development of uveitis.

### 2.4 OR11H1‐A63 Increased GADD45G Expression and Activated the NF‐κB and MAPK Pathways

**Figure 3:**
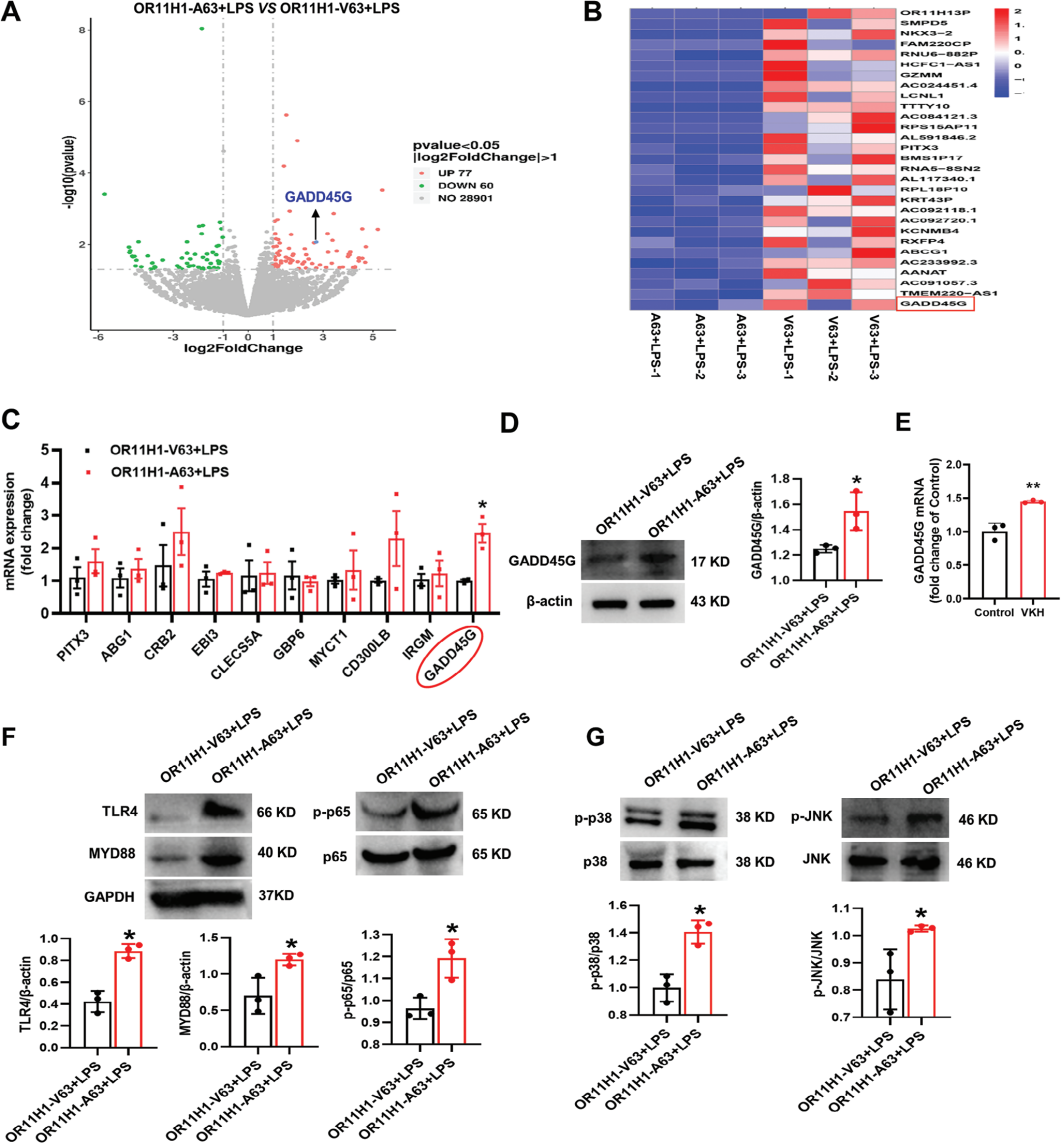
OR11H1‐A63 increased GADD45G expression and activated the NF‐κB and MAPK pathways. ARPE‐19 cells were randomly divided into the OR11H1‐V63 + LPS and OR11H1‐A63 + LPS groups. After incubation with LPS (1 µg ml^−1^) for 24 h, cells were collected. A) Volcano plots of RNA‐seq assays. Black arrows represent GADD45G. B) Heatmaps of RNA‐seq assays. Red boxes represent GADD45G. C) Through RNA‐seq assays, the expression levels of PITX3, ABG1, CRB2, EBI3, CLECS5A, GADD45G, MYCT1, CD300LB, IRGM and GBP6 were measured by RT‒qPCR. D) The protein expression of GADD45G was measured by Western blotting. E) Peripheral blood of VKH patients was collected and RNA was extracted for qPCR experiment. The expression of GADD45G was measured by RT‒qPCR. (F) The protein expression of TLR4, MYD88, p‐p65, and p65 was detected by Western blotting. G) The protein expression of p‐p38, p38, p‐JNK, and JNK was measured by Western blotting. ^*^ *P* < 0.05, ^**^ *P* < 0.01 by unpaired *t* test (n = 3).

To further determine the molecular mechanism underlying OR11H1‐A63 induced inflammation, we performed RNA‐seq assays in OR11H1‐A63 + LPS cells and OR11H1‐V63 + LPS cells. As shown in the volcano plots and heatmaps, 137 genes expression were considerable different between the two groups (**Figure** **3**A,B). According to RNA‐seq assays, some inflammation‐related genes, such as PITX3, ABG1, CRB2, EBI3, CLECS5A, and GADD45G were upregulated, while MYCT1, CD300LB, IRGM, and GBP6 were downregulated. In further validation, we discovered that only GADD45G exhibited a significant increase in mRNA expression in the OR11H1‐A63 + LPS group (Figure 3C). The protein expression levels of GADD45G were also significantly increased in the OR11H1‐A63 + LPS group (Figure 3D). The qPCR result showed that the expression of GADD45G was significantly increased in the peripheral blood mononuclear cells of VKH patients (Figure 3E). We therefore hypothesized that GADD45G may be involved in OR11H1‐A63 induced inflammation. We also measured the expression of proteins related to the NF‐κB pathway. As shown in Figure 3F, OR11H1‐A63 was found to increase protein expression of TLR4, MYD88 and p‐p65 (Figure 3F). Furthermore, several reports have noted that GADD45G can phosphorylate p38/MAPK and perform its function through the GADD45G/p38 MAPK pathway.^[^ ^40^ ^]^ We further analyzed whether the MAPK pathway participated in OR11H1‐A63 induced inflammation. Notably, the results revealed that OR11H1‐A63 elevated the expression of p‐p38 and p‐JNK (Figure 3G). Taken together, these results indicated that OR11H1‐A63 may regulate the NF‐κB and MAPK pathways in inflammation in ARPE‐19 cells.

### 2.5 GADD45G Knockdown Reversed OR11H1‐A63 Induced Inflammation

**Figure 4:**
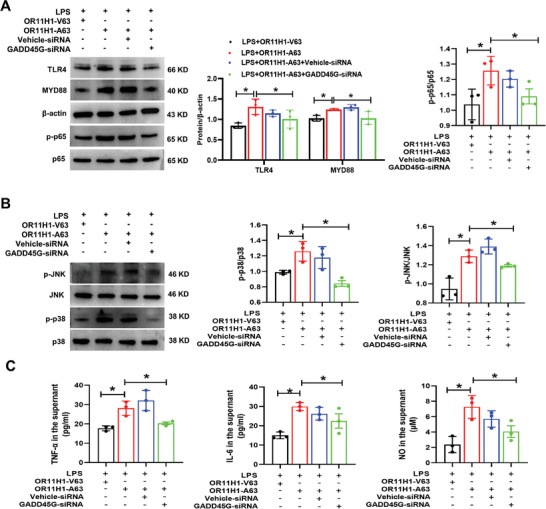
GADD45G knockdown reversed OR11H1‐A63 induced inflammation. ARPE‐19 cells were randomly divided into the OR11H1‐V63 + LPS, OR11H1‐A63 + LPS, OR11H1‐A63 + LPS + Vehicle‐siRNA, and OR11H1‐A63 + LPS + GADD45G‐siRNA groups. After stimulation with LPS (1 µg ml^−1^) for 24 h, the cells were collected. A) The protein expression of NF‐κB pathway components, such as TLR4, MYD88, p‐p65, and p65, was measured by Western blotting. B) The protein expression of MAPK pathway components, including p‐p38, p38, p‐JNK, and JNK, was measured by Western blotting. C) The levels of inflammatory factors, such as TNF‐α, IL‐6, and NO, were measured by ELISA and Griess reagent. ^*^ *P* < 0.05 by one‐way ANOVA (n = 3).

To determine whether OR11H1‐A63 regulates ARPE‐19 cell inflammation by increasing GADD45G expression, we knocked down GADD45G expression using siRNAs. We first evaluated the knockdown efficiency of three siRNAs by Western blotting. GADD45G‐siRNA3 exerted a significant inhibitory effect and was selected for subsequent experiments (Figure S4, Supporting Information). When GADD45G levels were depleted in ARPE‐19, the increased expression of TLR4, MYD88, p‐p65, p‐p38, and p‐JNK by OR11H1‐A63 were weakened (**Figure** **4**A,B). The results strongly implied that GADD45G was necessary for OR11H1‐A63 to activate the NF‐κB and MAPK pathways. In addition, the increased expression of TNF‐α, IL‐6, and NO by OR11H1‐A63 was reversed after blocking GADD45G expression (Figure 4C). In conclusion, these data suggested that OR11H1‐A63 induced an inflammatory response in a GADD45G‐dependent manner.

## 3 Discussion

In our study, we performed a WES analysis of VKH disease for the first time. The results revealed that a rare c.188T>C (p.Val63Ala) variant in the OR11H1 gene was significantly associated with VKH disease, indicating that this missense variant confers susceptibility to this disease. Next, functional study indicated that OR11H1‐A63 enhanced inflammatory factor expression and exacerbated barrier function damage in ARPE‐19 cells compared with OR11H1‐V63. In the subsequent RNA‐seq analysis of ARPE‐19 cells, we identified GADD45G as a downstream factor of OR11H1 and proposed a mechanism underlying the inflammatory cascade that functioned via dual inhibition of the MAPK and NF‐κB pathways.

Previous genetic studies have found significant association of non‐HLA genes such as IL23R and CTLA4 with VKH disease.^[^ ^41^, ^42^ ^]^ However, the discovered genes are mostly associated genes rather than causative genes of VKH disease. In this study, the OR11H1 rs71235604 (p.Val63Ala) gene was found to be the causative gene of VKH disease by WES and NGS analysis. This is the first application of WES to identify genetic variants of VKH disease that may contribute to fully understanding the pathological characteristics of this disease and may further provide new ideas and strategies for identifying the causative genes of other autoimmune diseases.

OR11H1 is a member of the OR family, and it belongs to the GPCR superfamily, which includes mostly favorable drug targets.^[^ ^30^, ^43^ ^]^ Recent studies have indicated that transplantation of olfactory ensheathing cells (OECs) plays pivotal roles in neural regeneration and functional recovery after neural injury, and OECs can promote neural repair by ameliorating overactive inflammatory responses.^[^ ^44^ ^]^ However, the function of ORs in devastating ocular inflammation is unknown. We identified that OR11H1‐A63 accelerated inflammatory factor release and BRB breakdown, suggesting that OR11H1‐A63 might be involved in the development of VKH disease, and could be considered a target for anti‐inflammatory treatments.

It is not clear which downstream targets and signaling pathways are activated in the acceleration of RPE‐induced inflammatory cascades in the context of OR11H1 mutation. RNA‐seq assays showed that OR11H1‐A63 significantly increased GADD45G expression. The qPCR results indicated that the GADD45G mRNA level was increased in VKH patients. GADD45G is a DNA damage response gene that further triggers an inflammatory cascade when the ability for DNA repair is impaired.^[^ ^40^, ^45^ ^]^ In addition to being a DNA damage‐responsive gene, GADD45G has also been shown to be involved in inflammation through p38 MAPK signaling.^[^ ^46^, ^47^ ^]^ Thus, GADD45G activation may account for the specific effects on inducing inflammation in RPE cells. Here, our data first identified that the OR11H1 missense variant activated the MAPK and NF‐κB pathways in a GADD45G‐dependent manner.

There are several limitations worth noting in this study. First, the patients and controls were enrolled from the Chinese Han population, and this study lack samples from other ethnic populations. Additionally, it was impossible to stratify the disease status and make the WES results more precise due to the limited size of VKH patients enrolled in this study. Second, we mainly focused on studying the association between non‐HLA gene mutations and VKH disease, and the function of HLA genes should be examined in further studies. Moreover, due to the difficulty of establishing HLA haplotype, we did not study its association with variant zygosity. Third, as OR11H1 is currently only found to be expressed in primates, this study did not conduct corresponding in vivo functional studies in EAU model. Additionally, further study is needed to investigate the expression and the function of OR11H1 in immune cells. Finally, since there are many inflammatory factors involved in VKH disease that have an impact and this study only focused on the most significant ones, such as TNF‐α, IL‐6.^[^ ^29^, ^48^, ^49^ ^]^ Therefore, further studies are needed to elucidate the roles of these variants in VKH disease and other uveitis entities in other ethnic populations.

In summary, our study found that OR11H1 missense variants played pivotal roles in the development of VKH disease. RNA‐seq results further showed that GADD45G was a major target of the OR11H1 missense variant. We further indicated that the MAPK and NF‐κB pathways were major signaling pathways that were activated in response to GADD45G upregulation and that induced ARPE‐19 cell inflammation. Moreover, GADD45G played a critical role in inhibiting inflammatory factors release.

## 4 Experimental Section

### Patient Recruitment

The present study was approved by the Ethics Committee of the First Affiliated Hospital of Chongqing Medical University (Permit Number: 2009–201008, 2019–296 and 2019–099). All the patients enrolled in this study were diagnosed as VKH disease by senior ophthalmologists according to the revised 2001 diagnostic criteria and those proposed by our group for VKH disease.^[^ ^50^, ^51^ ^]^ Controls were unrelated healthy individuals who had no family history of VKH disease and no autoimmune disorders. Written informed consent was obtained from all study participants. All VKH patients and controls enrolled in this study were registered with the China Human Genetic Resources Management Office (2021CJ2035). Genomic DNA was extracted from peripheral blood for genetic analysis following standard protocols.

### Whole‐Exome Sequencing

Agilent SureSelect Human All Exon V6 exome performs (Agilent, USA) captured the entire exome. After pooling, each captured library was sequenced for paired‐end 150 bp reads on the Illumina NovaSeq 6000 system (Illumina, USA). FastQC software was used to assess the quality of the raw data before adaptor sequencing, and poor‐quality fragments from the 3′‐end were excised and examined using the Trim Galore program. BWA software mapped the clean sequence fragments to the reference human genome, while Picard tools identified repetitions. Sentieon (sentieon‐genomics‐201611.02) was used to find sample variants, and ANNOVAR software was used to annotate sites based on genes, regions, and filters.

### Next‐Generation Sequencing

DNA extraction was performed using a Rapid Blood Genomic DNA Isolation Kit (Sangon, Shanghai, China). Paired‐end sequencing of the library was performed on the HiSeq XTen sequencers (Illumina, San Diego, CA). Raw reads were filtered according to two steps: 1) Removing adaptor sequence if reads contains by cutadapt (v 1.2.1); 2) Removing low‐quality bases from reads 3′ to 5′ (Q < 20) by PRINSEQ‐lite (v 0.20.3). The remaining clean data were mapped to the reference genome by BWA (version 0.7.13‐r1126) with default parameters. Samtools (Version: 0.1.18) was used to calculate each genotype of the target site. Annovar (2018‐04‐16) was used to detect genetic variants.

### ARPE‐19 Cell Line and Transfection

ARPE‐19 cells (ATCC, ATCC Number: CRL‐2302) were cultured in DMEM/F12 (1:1) supplemented with 10% fetal bovine serum in an incubator at 37°C in 5% CO_2._ The cells were seeded in 6‐well plates at 5 × 10^5^ cells/well for 24 h, and stimulated by LPS (1 µg/ml). The OR11H1‐A63 and OR11H1‐V63 lentiviruses were purchased from GeneChem (Shanghai, China). ARPE‐19 cells were transfected with OR11H1‐A63 (MOI = 30) or OR11H1‐V63 lentivirus (MOI = 30) according to the manufacturer's protocols and cultured with puromycin‐containing medium. GADD45G‐siRNA and Vehicle‐siRNA were synthesized by Sangon (Shanghai, China). GADD45G‐siRNA and Vehicle‐siRNA were transfected into ARPE‐19 cells with Lipofectamine 6000 for 8 h following the manufacturer's protocol. 24 h later, the transfected ARPE‐19 cells were observed under a confocal microscope and then collected for further analysis.

### RNA Sequence

TRIzol reagent (Invitrogen) was used to extract total RNA from ARPE‐19 cells. Agilent Bioanalyzer 2100 was used to verify RNA quality via agarose gel electrophoresis (RNA Integrity Number > 8). Transcriptome analysis was performed by Novogene Technology Co., Ltd. (Beijing, China). Differential expression analysis of two groups was performed using the DESeq2 R package. The Benjamini‒Hochberg method was used to control the false discovery rate. Genes with a P‐value <0.05 and |log2FoldChange|>1 found by DESeq2 were assigned as differentially expressed. Primer sequences are listed in Table S2 (Supporting Information).

### Western Blotting

ARPE‐19 cells were lysed in a lysis buffer to extract total protein, and the protein concentration was measured by a bicinchoninic acid assay kit (Beyotime, Shanghai, China). Samples were loaded and separated in 10% Bis‐Tris‐polyacrylamide electrophoresis gels. Then, the proteins were transferred to PVDF membranes, which were blocked with 5% nonfat milk followed by incubation with primary antibodies at 4 °C overnight. After incubation with the secondary antibody for 1 h, the Western blot signals were detected by ECL Plus reagents. ImageJ was used to analyze the protein bands. Primary antibodies against Occludin (1:800, Cat No. 17590‐1‐AP), TLR4 (1:800, Cat No. 66350‐1‐Ig), p65 (1:500, Cat No. 10745‐1‐AP), JNK (1:500, Cat No. 24164‐1‐AP), p‐JNK (1:500, Cat No. 80024‐1‐RR) and MYD88 (1:800, Cat No: 23230‐1‐AP) were purchased from Proteintech. Antibodies against p‐p65 (1:1000, AF2006), GADD45G (1:1000, A10286), p‐p38 (1:1000, AF4001), p38 (1:1000, AF6456), GAPDH (1:3000, AF7021), β‐actin (1:3000, AF7018) and OR11H1 (1:1000, DF5186) were purchased from Affinity.

### Real‐Time Quantitative PCR Analysis

RNA was extracted from ARPE‐19 cells using TRIzol reagent (Invitrogen). Real‐time quantitative PCR was performed with RT Master Mix and SYBR Green qPCR, which were obtained from MCE (Shanghai, China). GAPDH was used as the endogenous control, and the primer sequences of the target genes are shown in Table S2 (Supporting Information).

### Measurement of TNF‐α, IL‐6, and NO Levels

Supernatants were collected to analyze the levels of TNF‐α, IL‐6, and NO. TNF‐α and IL‐6 concentrations were measured by ELISA kits (R&D Systems), and NO levels were measured by Griess reagent according to the manufacturer's instructions.

### Quantification and Statistical Analysis

All data analysis for WES was completed by Novogene Bioinformatics Technology Co., Ltd. (Beijing, China). For the analysis of clinical samples, principal component analysis (PCA) was performed by projecting exome‐sequencing samples onto 1000 genome samples using Plink (v1.90b1g 64‐bit). Quantile‒quantile (Q‒Q) plot of single‐variant analyzes was generated from exome sequence data using the qqman R package (4.1.2). The Manhattan plot of single‐variant analyzes was generated from exome sequence data using the ggplot2 R package (4.1.2). The χ^2^ test was used to assess the Hardy‐Weinberg equilibrium of each tested SNP in the controls. SNPs were excluded from this analysis when they deviated from Hardy‐Weinberg equilibrium (HWE).^[^ ^52^ ^]^ The candidate SNP distributions of genotypes and frequencies of alleles were determined by χ^2^ test after weighting the data using SPSS 26.0, followed by the calculation of the odds ratio (OR) and 95% confidence interval (CI). For the validation test, SPSS 26.0 and GraphPad Prism software were used for statistical analysis and presentation, respectively. The data are presented as the mean ± SD. For statistical analysis, unpaired *t* tests were used to analyze the differences between two groups, and one‐way ANOVA was used to analyze the differences among multiple groups. ^*^ *P* < 0.05 was considered statistically significant.

## Conflict of Interest

The authors declare no conflict of interest.

## Author Contributions

X.L., G.W., X.W., and W.L. contributed equally to this work. XR.L and GQ.W completed this experiment; XT.W and WQ.L completed data analysis of clinical samples; PZ.Y, GN.S, QF.C, and SY.H participated in the collection of clinical samples; N.L, Y.H, XY. L and W.F. participated in data analysis of basic validation experiment. XR.L and GQ.W wrote this manuscript; PZ.Y and SP.H designed this study and revised the manuscript.

## Supporting information

Supporting Information

## Data Availability

Research data are not shared.
