# Supplementary material for: OR11H1 Missense Variant Confers the Susceptibility to Vogt‒Koyanagi‒Harada Disease by Mediating Gadd45g Expression
Source: Adv Sci (Weinh). 2024 Jan 2;11(11):2306563. doi: 10.1002/advs.202306563 (PMC10953539; doi:10.1002/advs.202306563)
Supplement: Supplementary file 1 — Supporting Information [file ADVS-11-2306563-s001.pdf]

ADVANCED SCIENCE
Open Access

Supporting Information

for *Adv. Sci.*, DOI 10.1002/advs.202306563

OR11H1 Missense Variant Confers the Susceptibility to Vogt–Koyanagi–Harada Disease by Mediating Gadd45g Expression

Xingran Li, Guoqing Wang, Xiaotang Wang, Wanqian Li, Na Li, Xianyang Liu, Wei Fan, Siyuan He, Yue Han, Guannan Su, Qingfeng Cao, Peizeng Yang and Shengping Hou**

Supporting information for

OR11H1 missense variant confers the susceptibility to Vogt–Koyanagi–Harada disease by mediating GADD45G expression

Xingran Li[1,2,3,4,#], Guoqing Wang[1,2,3,4,#], Xiaotang Wang[1,2,3,4,#], Wanqian Li[1,2,3,4,#], Na Li[5], Xianyang Liu[1,2,3,4], Wei Fan[1,2,3,4], Siyuan He[1,2,3,4], Yue Han[6], Guannan Su[1,2,3,4], Qingfeng Cao[1,2,3,4], Peizeng Yang[1,2,3,4,*], Shengping Hou[7,1,2,3,4]*

[1]The First Affiliated Hospital of Chongqing Medical University, Chongqing, China

[2]Chongqing Key Laboratory of Ophthalmology, Chongqing, China

[3]Chongqing Eye Institute, Chongqing, China

[4]Chongqing Branch of National Clinical Research Center for Ocular Diseases, Chongqing, China

[5]School of Basic Medical Sciences, Chongqing Medical University, Chongqing, 400016, China; Joint International Research Laboratory of Reproduction and Development, Chongqing Medical University, Chongqing, China

[6]Beijing Novogene Bioinformatics Technology Co.,Ltd, Beijing, China

[7]Beijing Institute of Ophthalmology, Beijing Tongren Eye Center, Beijing Tongren Hospital, Capital Medical University, Beijing Ophthalmology & Visual Sciences Key Laboratory, Beijing, 100730, China.

[#]These authors contributed equally

*Corresponding author: Professor Shengping Hou Ph.D, Beijing Institute of Ophthalmology, Beijing Tongren Hospital, Capital Medical University, Beijing, 100730, China; The First Affiliated Hospital of Chongqing Medical University, Chongqing 400016, China.

E-mail address: sphou828@163.com; Telephone number: 0086-10-58265906

& Professor Peizeng Yang, MD, Ph.D, The First Affiliated Hospital of Chongqing Medical University, Chongqing 400016, China

E-mail address: peizengycmu@126.com; Telephone number: 0086-23-89012851

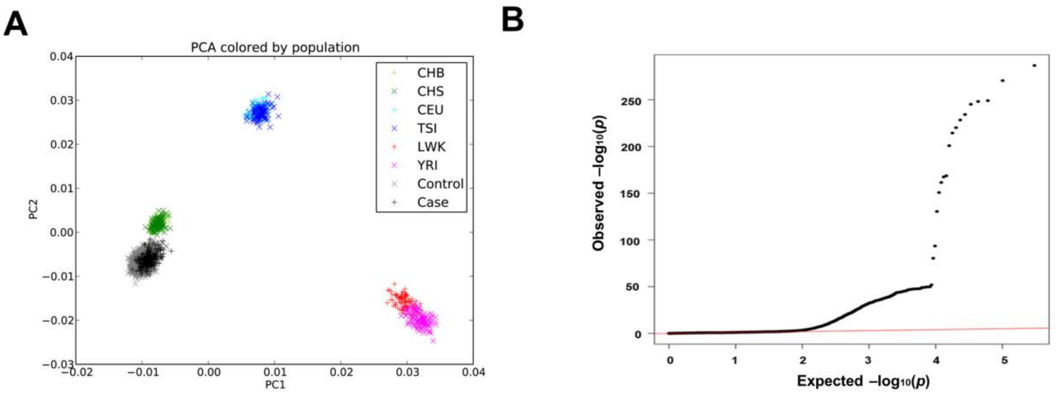

Figure S1 Whole-exome sequencing results for 229 cases with VKH syndrome and 2,573 controls from the Han Chinese population

(A) Ancestry analysis of the WES datasets using principal components. The x-axis shows eigenvector 1 (first principal component), and the y-axis shows eigenvector 2 (second principal component). (B) Quantile–quantile plot of the observed (y-axis) and expected (x-axis) P values from the WES association results for all SNPs.

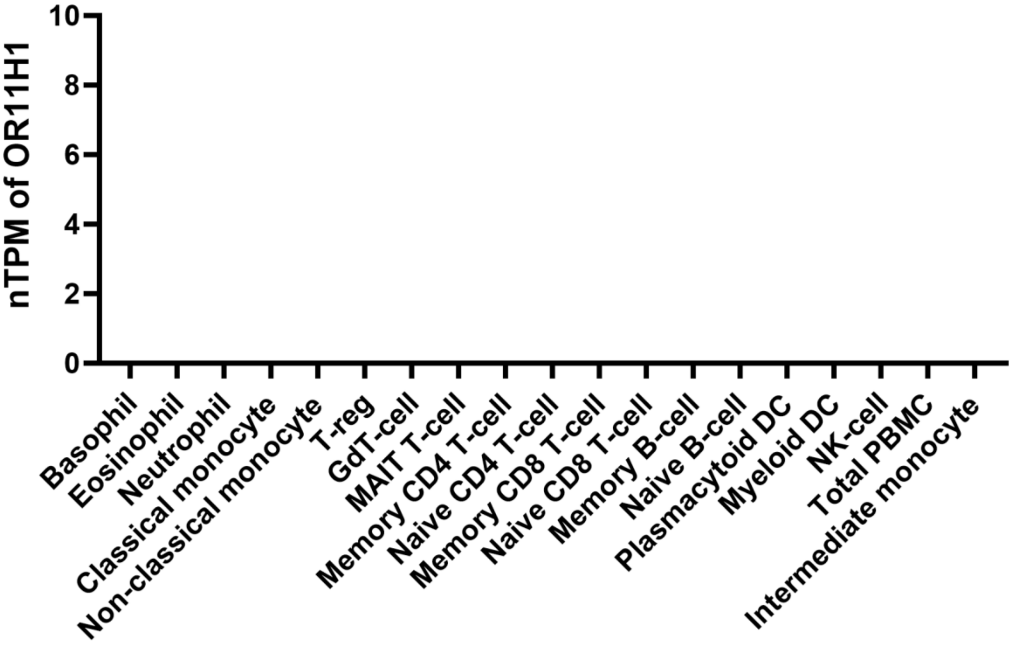

Figure S2 The expression of OR11H1

The nTPM (Transcripts Per Kilobase per Million mapped reads) of OR11H1 in immune cells from the Human Protein Atlas website.

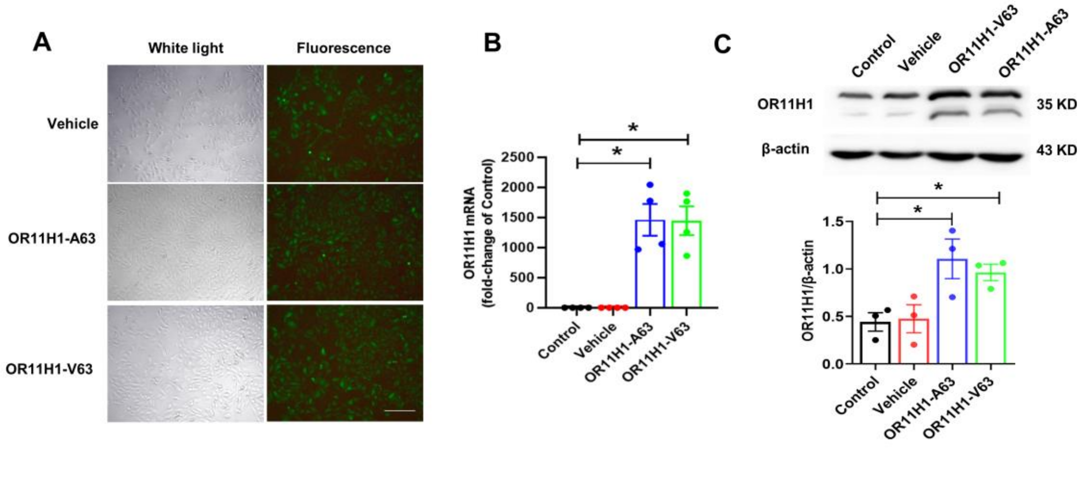

Figure S3 The overexpression efficiency of OR11H1

ARPE-19 cells were transfected with Vehicle, OR11H1-V63 and OR11H1-A63 lentivirus for 24 h, and the cells were randomly divided into the Control, Vehicle, OR11H1-A63 and OR11H1-V63 groups. (A) Transfection efficiency was observed by fluorescence microscopy. Scale bar = 100 μm. (B and C) OR11H1 overexpression efficiency was measured by RT–qPCR and Western blotting. *P<0.05 by one-way ANOVA (n = 3-4).

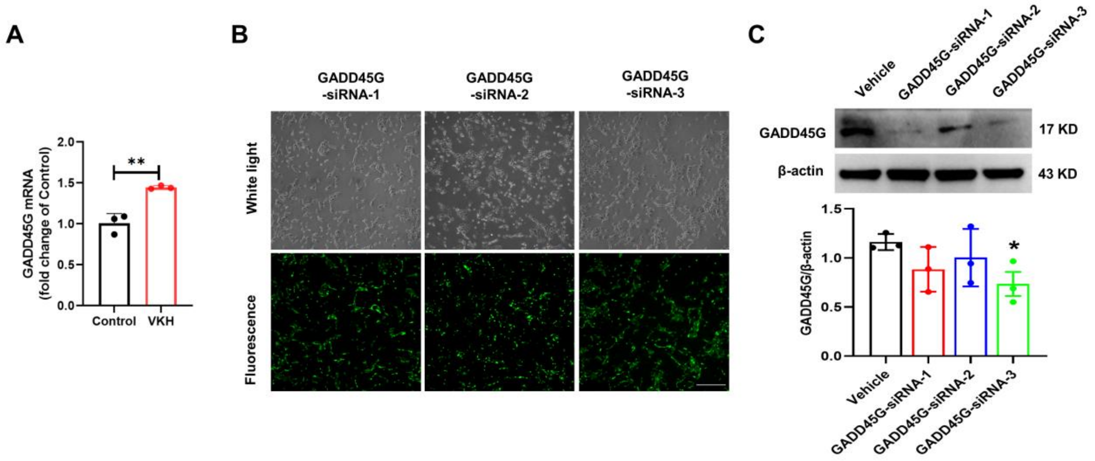

Figure S4 GADD45G knockdown efficiency

ARPE-19 cells were randomly divided into the Vehicle, GADD45G-siRNA-1, GADD45G-siRNA-2 and GADD45G-siRNA-3 groups for 24 h. (A) Transfection efficiency of GADD45G was observed by fluorescence microscopy. Scale bar = 100 μm. (B) Western blotting was used to analyse the GADD45G knockdown efficiency. *P<0.05，**P<0.01 (n = 3).

Table S1 Summary of the other SNPs with VKH disease in WES and replication studies

Genes	SNPs	Chr	MA	Stage	MAF (case/control)	HWE	*P*	OR	95%CI
NOTCH2	rs2603926	1	T	WES	0.075/0.024	0.4	4.19E-10	3.28	2.21-4.86
				Replication	0.106/0.108	5.09E-09	6.57E-01	0.97	0.85-1.11
				Combined	0.103/0.064		2.34E-16	1.66	1.47-1.88
OR2TB8	rs61857492	1	T	WES	0.104/0.026	1	6.41E-19	4.36	3.07-6.20
				Replication	0.008/0.003	1.39E-44	6.51E-02	2.27	0.93-5.58
				Combined	0.036/0.016		1.06E-07	2.32	1.69-3.20
NEB	rs62174690	2	G	WES	0.102/0.016	0.14	1.68E-21	6.95	4.38-11.01
				Replication	0.321/0.311	5.05E-04	2.93E-01	1.05	0.96-1.14
				Combined	0.309/0.226		1.15E-23	1.53	1.41-1.67
POTEI	rs200770323	2	A	WES	0.291/0.040	0.03	7.84E-17	3.49	2.56-4.77
				Replication	0.024/0.015	0.46	3.71E-03	1.58	1.16-2.15
				Combined	0.036/0.028		5.25E-02	1.22	1.00-1.49
EGFL8	rs2071289	6	A	WES	0.079/0.025	0.41	1.10E-10	3.29	2.25-4.82
				Replication	0.064/0.021	7.49E-05	6.16E-25	3.19	2.53-4.03
				Combined	0.066/0.023		1.34E-37	2.95	2.48-3.50
TNXB	rs3749960	6	T	WES	0.146/0.032	1	2.33E-31	5.11	3.78-6.91
				Replication	0.160/0.039	2.85E-05	1.51E-85	4.76	4.02-5.63

				Combined	0.159/0.035		5.11E-157	5.19	4.55-5.92
HLA-A	rs2916803	6	T	WES	0.478/0.028	0.14	4.02E-307	31.8	24.83-40.73
				Replication	0.021/0.035	2.66E-25	3.78E-05	0.59	0.45-0.76
				Combined	0.063/0.031		1.29E-19	2.08	1.77-2.44
HLA-B	rs1130992	6	C	WES	0.564/0.280	1	2.31E-36	3.32	2.73-4.04
				Replication	0.280/0.274	0.43	5.68E-01	1.03	0.94-1.12
				Combined	0.301/0.277		3.67E-04	1.15	1.06-1.23
HLA-C	rs35708511	6	C	WES	0.892/0.172	0.02	9.41E-267	39.69	29.25-53.85
				Replication	0.185/0.171	0.21	9.14E-02	1.1	0.99-1.22
				Combined	0.249/0.172		4.96E-29	1.6	1.47-1.74
HLA-DPA1	rs1126542	6	T	WES	0.923/0.359	1.53E-03	3.05E-122	21.45	15.13-30.43
				Replication	0.214/0.350	0.64	1.58E-47	0.51	0.46-0.56
				Combined	0.279/0.355		1.08E-20	0.7	0.65-0.76
HLA-DPA1	rs114707319	6	G	WES	0.982/0.362	1.36E-03	4.11E-146	98.24	48.72-198.12
				Replication	0.214/0.350	0.64	1.89E-47	0.51	0.46-0.56
				Combined	0.284/0.356		8.71E-19	0.72	0.67-0.77
HLA-DPA1	rs2308912	6	A	WES	0.876/0.382	9.61E-03	1.68E-89	11.44	8.55-15.29
				Replication	0.223/0.367	1.61E-05	1.20E-51	0.5	0.45-0.54
				Combined	0.280/0.375		2.34E-30	0.65	0.60-0.70
HLA-DPA1	rs34950776	6	C	WES	0.988/0.359	1.76E-03	4.85E-144	152.28	62.94-368.41
				Replication	0.213/0.350	0.68	2.92E-48	0.5	0.46-0.55

				Combined	0.280/0.355		8.62E-20	0.71	0.66-0.76
HLA-DPB1	rs1042121	6	C	WES	0.903/0.159	1	1.16E-290	49.34	35.71-68.17
				Replication	0.113/0.155	0.05	2.79E-09	0.69	0.61-0.78
				Combined	0.184/0.157		4.07E-05	1.21	1.10-1.32
HLA-DQA1	rs7990	6	A	WES	0.058/0.215	0.09	1.45E-15	0.22	0.15-0.33
				Replication	0.518/0.229	0.74	1.03E-183	3.63	3.32-3.97
				Combined	0.477/0.221		1.91E-223	3.2	2.98-3.45
HLA-DQA1	rs9272756	6	T	WES	0.982/0.390	0.06	2.43E-127	84.34	41.82-170.11
				Replication	0.219/0.395	0.3	9.92E-76	0.43	0.39-0.47
				Combined	0.286/0.399		1.07E-37	0.62	0.58-0.67
HLA-DQA2	rs2051599	6	T	WES	0.941/0.246	0.79	5.51E-210	48.59	32.77-72.06
				Replication	0.179/0.250	0.3	3.83E-17	0.65	0.59-0.72
				Combined	0.248/0.248		9.55E-01	1	0.92-1.08
HLA-DQA2	rs2051600	6	A	WES	0.336/0.078	0.41	2.41E-69	5.94	4.77-7.40
				Replication	0.055/0.081	0.51	6.90E-07	0.66	0.56-0.78
				Combined	0.081/0.080		8.28E-01	1.01	0.89-1.15
HLA-DQB1	rs1130398	6	C	WES	0.590/0.504	0.97	1.58E-08	1.77	1.45-2.17
				Replication	0.213/0.476	9.54E-242	6.71E-156	0.3	0.27-0.33
				Combined	0.252/0.491		2.16E-172	0.35	0.32-0.38
HLA-DQB2	rs3213486	6	T	WES	0.929/0.166	0.52	7.17E-300	65.56	45.42-94.62
				Replication	0.115/0.169	0.24	1.41E-13	0.64	0.57-0.72

				Combined	0.189/0.167		1.43E-03	1.15	1.06-1.26
HLA-DQB2	rs3213487	6	C	WES	0.958/0.166	0.52	2.23E-319	113.32	71.16-180.46
				Replication	0.115/0.169	0.23	1.62E-13	0.64	0.57-0.72
				Combined	0.191/0.167		4.13E-04	1.17	1.07-1.28
HLA-DQB2	rs3213489	6	T	WES	0.921/0.166	0.52	2.33E-296	58.35	41.19-82.67
				Replication	0.115/0.169	0.27	9.40E-14	0.64	0.57-0.72
				Combined	0.188/0.168		1.63E-03	1.15	1.06-1.26
HLA-DRB1	rs2308759	6	T	WES	0.942/0.193	0.66	8.04E-261	68.2	45.27-102.75
				Replication	0.149/0.168	0.04	1.10E-02	0.86	0.77-0.97
				Combined	0.157/0.034		1.21E-156	5.24	4.59-5.98
HLA-DPB1	rs707958	6	C	WES	0.971/0.159	0.94	2.63E-34	175.93	100.81-307.00
				Replication	0.110/0.158	4.58E-04	2.48E-11	0.66	0.59-0.75
				Combined	0.187/0.158		7.62E-06	1.23	1.12-1.34
HLA-DRB1	rs9270299	6	A	WES	0.939/0.160	0.51	3.13E-303	80.3	53.63-120.24
				Replication	0.204/0.172	1.49E-126	7.16E-05	1.24	1.11-1.37
				Combined	0.266/0.166		1.08E-47	1.83	1.69-1.99
HLA-DRB5	rs41546317	6	C	WES	0.008/0.221	0.91	1.41E-23	0.03	0.01-0.09
				Replication	0.090/0.150	5.56E-122	6.36E-19	0.56	0.49-0.64
				Combined	0.083/0.187		2.84E-61	0.4	0.35-0.44
MUC3A	rs35212402	7	T	WES	0.002/0.048	3.89E-03	4.41E-06	0.04	0.01-0.31
				Replication	0.087/0.079	2.90E-05	2.03E-01	1.1	0.95-1.28

				Combined	0.079/0.038		3.39E-04	1.27	1.11-1.45
PDPR	rs200469748	16	G	WES	0.140/0.016	1	3.52E-57	10.07	7.14-14.20
				Replication	0.000/0.001	0.94	3.90E-02	0.15	0.02-1.21
				Combined	0.013/0.009		1.98E-02	1.46	1.06-2.02
CLEC18B	rs199633934	16	T	WES	0.053/0.003	1	1.95E-34	19.75	10.14-38.47
				Replication	0.029/0.029	0.02	8.75E-01	1.02	0.80-1.30
				Combined	0.032/0.015		1.09E-10	2.08	1.66-2.61
GPR32	rs201404376	19	C	WES	0.031/0.002	1	2.39E-18	14.69	6.63-32.54
				Replication	0.000/0.001	0.98	3.33E-01	0.34	0.04-3.31
				Combined	0.003/0.001		4.05E-02	2.11	1.02-4.37
MADCAM1	rs77264553	19	A	WES	0.132/0.041	0.02	1.16E-17	3.52	2.59-4.77
				Replication	0.000/0.001	1.64E-129	1.92E-01	0.26	0.03-2.31
				Combined	0.012/0.022		5.19E-05	0.56	0.42-0.74

Chr., chromosome; MA, minor allele; MAF, minor allele frequency; HWE, Hardy-Weinberg Equilibrium result; OR: odds ratio for the minor allele; 95% CI, 95% confidence intervals.

Table S2 The primer sequences of targeted genes

Gene	Source	Forward (5'-3')	Reverse (5'-3')
OR11H1	Human	GGGTCTCATTTGGCTGTGGTATC	ATCCCTGTAGAATGTCCGAGTCC
PITX3	Human	CAGAGGACGGTTCGCTGAAAA	AGCTGCCTTTGCATAGCTCG
ABG1	Human	GGGGTCGCTCCATCATTTG	TTCCCCGGTACACACATTGTC
CRB2	Human	TCTCGGTGAGAACGTCCTCC	ATGAGTCCACAGATCCACACA
EBI3	Human	TCATTGCCACGTACAGGCTC	GGGTCGGGCTTGATGATGTG
CLECS5A	Human	AGGTGGCGTTGGATCAACAA	TTAGGCCAATGGTCGCACAG
GBP6	Human	AACCATCTGGCAGGACAGAAT	TCACCCTTTTCCACATCGCC
MYCT1	Human	GTGAACGTCGAAGCAACCTCA	ACTCTCAGTTTCCACAGGAAGT
CD300LB	Human	GGTCCCTGACGGTTCAATG	GATGGACACACGGTCACTCTT
IRGM	Human	CCTCACCTCCTACTGAGCTG	GTTTTGGCAAGCATCACATGAT
GADD45G	Human	CAGATCCATTTTACGCTGATCCA	TCCTCGCAAAACAGGCTGAG
